# The other face of miR-17-92a cluster, exhibiting tumor suppressor effects in prostate cancer

**DOI:** 10.18632/oncotarget.12061

**Published:** 2016-09-16

**Authors:** Richard Ottman, Jenna Levy, William E. Grizzle, Ratna Chakrabarti

**Affiliations:** ^1^ Burnett School of Biomedical Sciences, University of Central Florida, Orlando, Florida, USA; ^2^ Department of Pathology, University of Alabama at Birmingham, Birmingham, Alabama, USA

**Keywords:** miR-17-92a cluster, prostate cancer, tumor suppressor

## Abstract

miR-17-92a cluster miRNAs are transcribed from a polycistronic transcription unit C13orf25 that generates six mature miRNAs, miR-17, miR-18a, miR-19a, miR-19b, miR-20a and miR-92a that are overexpressed in lung and colon cancers. Here we show that the expression of miR-17-92a miRNAs are reduced in cancerous prostate tissues compared to uninvolved areas and also in aggressive prostate cancer cells. Restoration of expression of all members of miR-17-92a cluster showed, decreased expression of cell cycle regulatory proteins cyclin D1 and SSH1; and LIMK1 and FGD4 of RhoGTPase signaling pathway. Expression of miR-17-92a miRNAs caused decreased cell proliferation, reduced activation of AKT and MAP kinases, delayed tumorigenicity and reduced tumor growth in animals. Expression of miR-17-92a miRNAs inhibited EMT via reduced cell migration and expression of mesenchymal markers while elevating expression and surface localization of the epithelial marker E-Cadherin. Expression of miR-17-92a miRNAs improved sensitivity of androgen dependent LNCaP 104-S prostate cancer cells to anti-androgen drug Casodex, AKT inhibitor MK-2206 2HCl, and docetaxel. The androgen refractory PC-3 cells also showed increased sensitivity to docetaxel, MK-2206 2HCl and Aurora kinase inhibitor VX680 upon ectopic expression of miR-17-92a cluster miRNAs. Our data demonstrate a tumor suppressor effect of miR-17-92a cluster miRNAs in prostate cancer cells and restoration of expression of these miRNAs has a therapeutic benefit for both androgen-dependent and -independent prostate cancer cells.

## INTRODUCTION

MicroRNAs (miRNA) are recognized as a group of small noncoding RNAs that play diverse roles in regulation of biological processes and have a unique expression profile that varies in a tissue and disease specific manner [1, 2]. Expression of miRNAs is often deregulated in early stage cancers, which facilitates development of aggressive and drug resistant disease [3, 4]. MicroRNAs are often designated as oncogenic miRNAs or tumor suppressor miRNAs based on the expression patterns and functions [5, 6]. However, emerging evidence indicates that miRNA expression and functions are in most cases context specific and are influenced by other regulatory agents [7]; therefore, it is not unusual to encounter tumor suppressor effects of miRNAs that show oncogenic functions in other specific cancers [8].

miR-17-92a cluster miRNAs are transcribed from the polycistronic miR-17-92a gene located in the third intron of a primary transcript C13orf25 [9] that is processed into six mature miRNAs viz. miR-17, miR-18a, miR-19a, miR-19b, miR-20a and miR-92a [10]. miR-17-92a cluster has been differentially expressed in various cancers [11–13] and based on the function of the target genes, has been designated as oncogenic miRNAs [14, 15]. In neuroblastoma cells, the miR-17-92a cluster has been shown to impair TGF-β signaling pathway through miR-17 and −20a targeting of SMAD2, SMAD4 and transforming growth factor beta receptor 2 (TGFBR2) [16]. Expression of miR-17-92a cluster facilitates cell growth by reducing hypoxia inducible factor 1 α (HIF-1α) activity in lung cancer cells [17] and promotes cell survival through miR-19 mediated targeting of PTEN [18]. However, published reports also show anti-proliferative and senescence promoting effects of the members of the miR-17-92a clusters through direct targeting of Dicer by miR-18a in bladder cancer cells [19]; through down-regulation of the Amplified In Breast cancer 1 (AIB) mRNAs by miR-17-5p [20]; and through destabilization of proto-oncogene Leukemia Related Factor (LRF) mRNA by miR-20a [21]. These effects further emphasize the contextual aspects of the functional complexities of miR-17-92a cluster. Loss of expression of miR-17-3p has been noted in prostate cancer [22] and a functional relationship between the members of miR-17-92a cluster and androgen receptor function has been speculated [10, 23]. However, in depth understanding of the function of the miR-17-92a cluster miRNAs in prostate cancer growth and drug resistance is lacking.

Prostate cancer affects a significant number of men in developed countries where it is typically the second leading cause of cancer related deaths [24]. Although initially slow growing, prostate cancer can advance rapidly, leading to development of metastatic cancers [25, 26]. Surgical therapy is commonly used for localized disease but androgen deprivation therapy (ADT)/anti-androgen treatment is the front line therapy upon relapse [27, 28]. Nonetheless, development of ADT/anti-androgen resistance is not uncommon, which leads to generation of castration resistant prostate cancer (CRPC) [29, 30]. Several miRNAs are known to be involved in progression of localized prostate cancer to metastasis including miRs-let7c, −143, −145 and −218 [31, 32]. Mavridis *et al* showed loss of miR-224 expression in advanced prostate cancer and that sustained miR-224 expression is associated with favorable prognosis [33]. Loss of expression of miRs-205, −34b/c and −302a, which target Bcl2 and AKT, has been documented in high-grade prostate cancers [34–37], whereas, up-regulation of miRNAs including miRs-183, −153, and −125b, which target SMAD4, PTEN, p53, Puma and Bak1 has been noted in aggressive prostate cancers [38–40]. Nonetheless, most of the functional studies are on individual miRNAs, which may not represent the true environmental milieu of gene regulation, because miRNAs often function as part of a regulatory network.

Earlier, we showed loss of expressions of the members of miR-17-92a cluster as prostate cancer cells progressed to antiandrogen resistance [41]. In this study, we investigated the expression of miR-17-92a cluster in prostate tissues, its role in destabilization of mRNA targets such as cyclin D1, FGD4, LIMK1 and Slingshot phosphatase (SSH1) and its potential effects on activation of signaling cascades, tumor growth and drug sensitivity using cell culture, and xenograft models. These data demonstrate anti-oncogenic and drug-sensitivity promoting functions of miR-17-92a cluster miRNAs when ectopically expressed in prostate cancer cells.

## RESULTS

### Loss of expression of miR-17-92a cluster in prostate tumor tissues and cells

**Figure 1:**
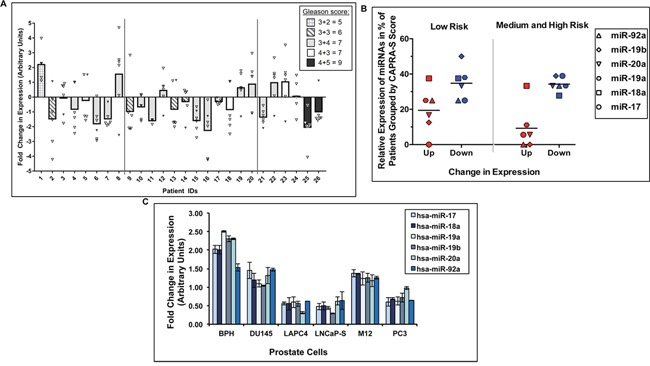
Expression profiles of the miR-17-92a cluster miRNAs in clinical samples and cell lines Quantitative real-time PCR showing relative expression of miR-17, miR-18a, miR-19a, miR-20a, miR-19b, and miR-92a. Raw data have been normalized to the average of 3 reference genes (RNU43, U6 snRNA, U1 snRNA). **A.** Average fold change in expressionof entire miR-17-92a cluster (Bars) in patient tumors compared to matched uninvolved adjacent tissues. Open triangles denote the fold change values of miR-17, miR-18a, miR-19a, miR-19b and miR-20a. Filled triangles denote values of miR-92a. **B.** Dot plots displaying the percentage of patients having fold change values of ≥ 1.5 for each miRNA. Horizontal bars designate the average value for the cluster. Red and blue symbols indicate up- or down- regulation, respectively. Patients were separated into two risk groups based on CAPRA-S score (Low=≤2, Medium and High=≥3. All values for A and B are an average of two separate analyses. *p*= 0.02 (upregulated miRNAs in risk group ≤2 vs. upregulated miRNAs in risk group ≥3, except miR-18a) *p*=0.04 (upregulated miRNAs vs. down regulated miRNAs in cases with CAPRA-S score ≤2), *p*=0.0009 (upregulated miRNAs vs. down regulated miRNAs in cases with CAPRA-S score ≥3) **C.** Fold change in expression of miR-17-92a cluster miRNAs in benign and cancerous prostate cell lines. BPH: BPH-1, LNCaP-S: LNCaP 104-S cells. Data show average of three separate analyses.

Our studies on genome-wide profiling of miRNAs using LNCaP cell culture model showed down-regulation of miR-17-92a cluster in anti-androgen resistant cells [41]. In this study, validation of expression profiles in clinical specimens also showed loss of expression of this cluster miRNAs. We used macro-dissected prostate tumor tissues and corresponding uninvolved areas to monitor expression of mature miR-17, −18a, −20a, −19a, −19b and −92a miRNAs. Patients were selected based on specific criteria including no prior treatments, Gleason Scores, pre-surgical PSA and local invasion; and based on CAPRA-S score [42] stratified into low, medium and high risk of biochemical recurrence (Table 1). Normalized fold-change (FC) expression analysis showed a distinct down-regulation/loss of expression of all members of the miRNA cluster in 58-73% of the cases tested (Figure 1A and Supplementary Table S1). Comparative analysis of the expression data revealed that: A) the average expression of all miRNAs was reduced in the majority of the cases with Gleason Scores between 6-9 including two cases with Gleason Scores of 9, and B) an increasing percentage of cases from low to high risks groups showed reduced expression of miR-92a (37% of low risk, 75% of medium risk and 83% of high risk) (solid triangle Figure 1A). Correlative analyses of the miRNA expression with at least a 1.5-fold change in expression and risk assessment showed an average down-regulation of the cluster in 35% of low risk cases (CAPRA-S≤2) and an average up-regulation in 19% of the cases. For patients with a higher risk (CAPRA-S≥3), the percentage of patients with down-regulated miR-17-92a miRNAs showed no change at 34% (Figure 1B); however, a distinct reduction in the average percentage of patients with up-regulation at only 9% for these miRNAs could be noted. Additionally, no patients with CAPRA-S ≥3 displayed increased expression of miR-19b or miR-92a (Figure 1B). Further correlation analysis of expression and CAPRA-S risk scores showed that four, five or all miRNAs were down-regulated in 67% of the cases in the high-risk and medium-risk groups (Table 2). Reduced expression of three, two or one miRNAs was noted in the rest of the 33% cases in the high or medium-risk groups. In the low risk group, four, five or all miRNAs were down-regulated in 50% of the cases while loss of one, two or three miRNAs were noted in the other 50% of the cases. Analysis of endogenous expression of these miRNAs in prostate adenocarcinoma and BPH-1 cell lines also showed reduced expression of most of the miRNAs in cancer cells compared to BPH-1 cells (Figure 1C). This observation led to the subsequent experiments to understand the functional significance of the loss of expression of miR-17-92a cluster in phenotypic changes in prostate cancer cells.

**Table 1:** Patient criteria and assessment of the risk of recurrence

Patient #	PSA pre-surgery	Gleason Score	CAPRA-S Score	Risk
1	14.3	3+2=5(IV)	2	low
2	5.9	3+3=6	2	low
3	4.3	3+3=6	2	low
4	8.2	3+4=7	2	low
5	7.8	3+4=7	2	low
6	8.8	3+4=7	2	low
7	6.6	3+4=7	2	low
8	3.7	3+4=7	2	low
9	23.3	3+3=6	3	medium
10	6.3	3+4=7	3	medium
11	6.2	3+4=7	3	medium
12	4.7	3+4=7	3	medium
13	5.1	3+3=6	4	medium
14	87.4	3+3=6	4	medium
15	9.8	3+4=7	4	medium
16	9.4	3+4=7	4	medium
17	8.8	3+4=7	4	medium
18	5.4	4+3=7	4	medium
19	6.5	3+4=7	5	medium
20	6.5	3+4=7	5	medium
21	4.8	3+4=7	7	high
22	14.9	3+4=7	7	high
23	13.9	4+3=7	7	high
24	5.6	4+3=7	8	high
25	31.8	4+4=8	9	high
26	51.8	4+5=9	9	high

**Table 2:** Correlative analysis of down regulation of miRNA expression with risk of recurrence

Risk	All miRNAs	Five miRNAs	Four miRNAs	Three miRNAs	Two/One miRNAs
High (6)	2	1 (miR-17, −18a, −20a, −19b, −19a	1 (miR-18a, −19a, −20a and −92a)	0	2 (miR-92a)
Medium (12)	4	1 (miR-17, −19a, −19b, −20a, −92a)	3 (miR-17/or-18a, −19a, −19b/or −20a/or −92a)	1 (miR-18a, −19b, −20a)	3 (miR-92a, and −20a)
Low (8)	1	2 (miR-17, −19a, 19b, −20a, −18a/ or −92a)	1 (miR-17, −18a, −20a, −92a)	1 (miR-19a, −17/or −18a/ or −19b/or −20a)	3 (miR-18a/ −19b/ or −92a)

### Expression of miR-17-92a cluster altered cell morphology and reduced expression of actin cytoskeleton modulatory and cell cycle regulatory proteins

**Figure 2:**
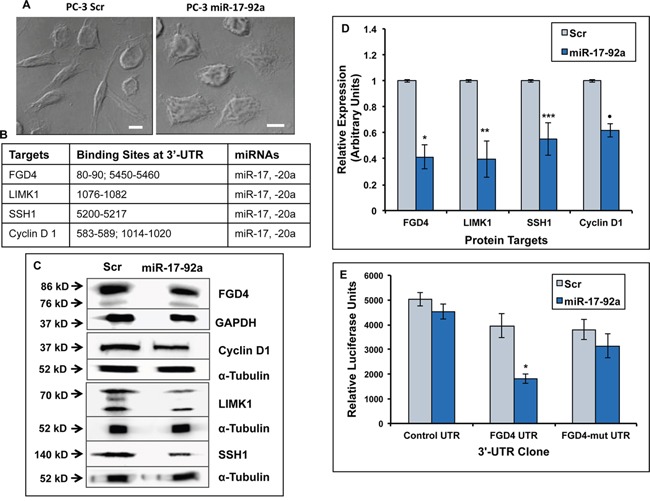
Expression of miR-17-92a cluster in prostate cancer cells altered cellular morphology and reduced expression of the putative target genes **A.** Bright field phase contrast images of PC-3 cells expressing miR-17-92a cluster miRNAs or Scr RNA. Scr RNA expressing cells retain parental PC-3 cell morphology (Left), whereas stable expression of miR-17-92a cluster miRNAs induced a more adherent phenotype and reduced the number of spindle-like cells (Right) Scale bar: 10μm. **B.** Predicted binding sites of miR-17, and miR-20a in the 3′UTRs of the target mRNAs **C.** Representative images of immunoblots of FGD4, cyclin D1, LIMK1, and SSH1 in PC-3 cell lysates showing reduced expression of all of these predicted targets upon expression of miR-17-92a cluster miRNAs. GAPDH and α-tubulin were used as the loading controls. **D.** Densitometric analysis of protein concentrations normalized to the internal controls. Data shows mean±SD of three independent analyses. *p=0.0004, **p=0.0004; ***p=0.003 ·*p* <0.0001. **E.** Luciferase reporter assays confirming that miR-17a and −20a directly target FGD4 through binding to the specified seed sequence at the 3′UTR. PC-3 cells were co-transfected with the luciferase constructs containing a nonspecific DNA sequence, a fragment of wild type FGD4 3′ UTR or a fragment of FGD4 3′ UTR containing mutations at the miR-17/20a seed sequence along with a plasmid expressing either miR-17-92a miRNAs or scrambled RNA. Data represent the mean±SD of three independent experiments. * *p*=0.001.

We generated sublines of PC-3 prostate cancer cells that overexpress all mature miRNAs of the miR-17-92a cluster compared to the control cells expressing scrambled (Scr) RNA upon transfection with the precursor miRNAs (Supplementary Figure S1A). The extent of expression was increased between 1.3-2.5-fold, a level comparable to that was detected in BPH-1 cells. We intended to express all miRNAs in the cluster simultaneously instead of single miRNAs to mimic the physiological condition and study the combinatorial effects. Overexpression of all miRNAs changed the morphology of PC-3 cells from spindle shapes to a more adherent cobblestone type (Figure 2A). Expression of these miRNAs resulted in 25%-40% reduction in concentration of some of the computer algorithm based predicted target proteins FGD4, LIMK1, SSH1 and cyclin D1, which contain one or more binding site(s) of miR-17 and −20a but none of the other miRNAs of the cluster, at the 3′-UTR (Figure 2B). PC-3 cells were used to confirm the direct binding of miR-17 and −20a to the predicted sites of one of the targets, FGD4. Luciferase reporter assays were done upon transient co-transfection of the miR-17-92a precursor construct plus a luciferase construct containing a fragment (bases from position 80-90) of FGD4 3′-UTR, in PC-3 cells. Results showed 54% inhibition of translation of FGD4 mRNA, which could be rescued by mutating miR-17 and −20a binding sites at the 3′-UTR (Figure 2E). Additionally, we validated destabilization of the 3′-UTRs of LIMK1 and SSH1 using luciferase reporter assays upon co-transfection of LIMK1 and SSH1 luciferase constructs along with miR-17-92a expression vectors, (Supplementary Figure S2), which showed 47% and 26% reduction in luciferase activities, respectively. Binding of miR-17 and −20a at the 3-UTR of cyclin D1 has already been experimentally validated [43]. Taken together, these results confirmed that overexpression of miR-17-92a reduced synthesis of proteins involved in activation of RhoGTPase pathway (FGD4) [44], actin cytoskeleton reorganization (LIMK1) [45, 46] and cell cycle regulation (SSH1, cyclin D1) that are often hyper-activated or over-expressed in advanced prostate cancer [47–49].

### Expression of miR-17-92a cluster inhibited activation of MAP kinase and AKT pathways and reduced cell proliferation

**Figure 3:**
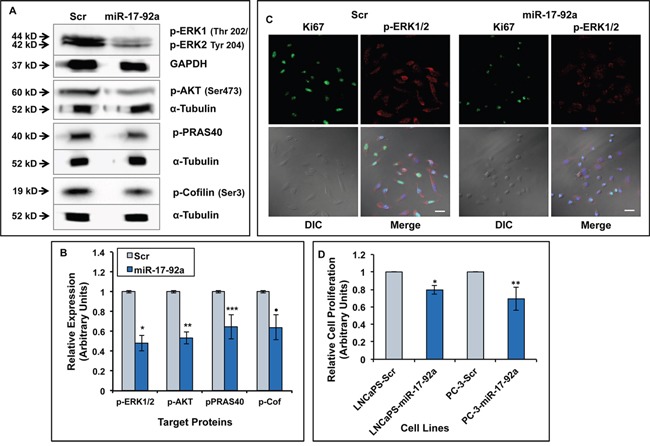
Expression of miR-17-92a miRNAs reduced activation of MAPK and AKT pathways and decreased cell proliferation **A.** Immunoblot analysis showing reduced phosphorylated ERK1/ERK2, -AKT, -PRAS40 (AKT substrate), and -Cofilin (LIMK1 substrate) in lysates from PC-3 cells stably expressing miR-17-92a cluster compared to cells expressing Scr RNAs. GAPDH or α-tubulin was used as the loading controls **B.** Densitometric analysis of the phosphoprotein concentrations normalized to internal controls. Data represent mean±SD of three separate experiments. *p=0.0003, **p=0.0001, ***p=0.007, ^·^p=0.008. **C.** Immunofluorescence analysis showing reduced expression ofKi-67 proliferation marker and phosphorylated ERK1/2 in PC-3 cells transiently expressing miR-17-92a miRNAs compared to control cells. Scale bar: 25μm. **D.** Cell proliferation assay showing reduced cell growth in miR-17-92a cluster miRNA expressing cells. LNCaP 104-S (LNCaPS) or PC-3 cells were transiently transfected with DNA constructs for Scr RNA or miR-17-92a precursor miRNAs and cell proliferation at 72 hr were detected by MTS assays. Data represent mean±SD of six independent experiments in triplicates. *p<0.0001; **p=0.0001.

Our results showing inhibition of expression of cyclin D1 prompted us to study cell proliferation and activation of the signaling pathways that promote cell proliferation. Using western blots, we detected expression of phosphorylated ERK1/2, phosphorylated AKT and phosphorylated AKT substrate, PRAS40, as markers for activation of MAP Kinase and AKT pathways in whole cell lysates of PC-3 cells expressing miR-17-92a cluster miRNAs. Our analyses showed a significant reduction (40%-55%) of these proteins in cells expressing miR-17-92a miRNAs compared to control cells expressing Scr RNAs (Figure 3A and 3B and Supplementary Figure S3). Although a slight reduction (20%) in total AKT was noted the reduction in phosphorylated AKT was much higher in miR-17-92a miRNA cluster expressing cells. No changes in the expression of total ERK and PRAS40 were noted in these cells (Supplementary Figure S3). A reduced expression of p-ERK1/2 in miR-17-92a miRNAs expressing cells as detected by immunofluorescence analysis, confirmed the western blot data (Figure 3C). The percentage of cells expressing Ki-67 antigen, a cell proliferation marker [50], was also reduced significantly in transiently transfected PC-3 cells upon expression of miR-17-92a (63% vs. 83%) compared to Scr RNA expression as detected by immunofluorescence (Figure 3C). Comparative MTS assays showed a significantly reduced (20%-30%) growth of transiently transfected LNCaP 104-S and PC-3 cells upon expression of miR-17-92a miRNAs (Figure 3D).

### Expression of miR-17-92a cluster delayed tumorigenicity and reduced tumor growth

**Figure 4:**
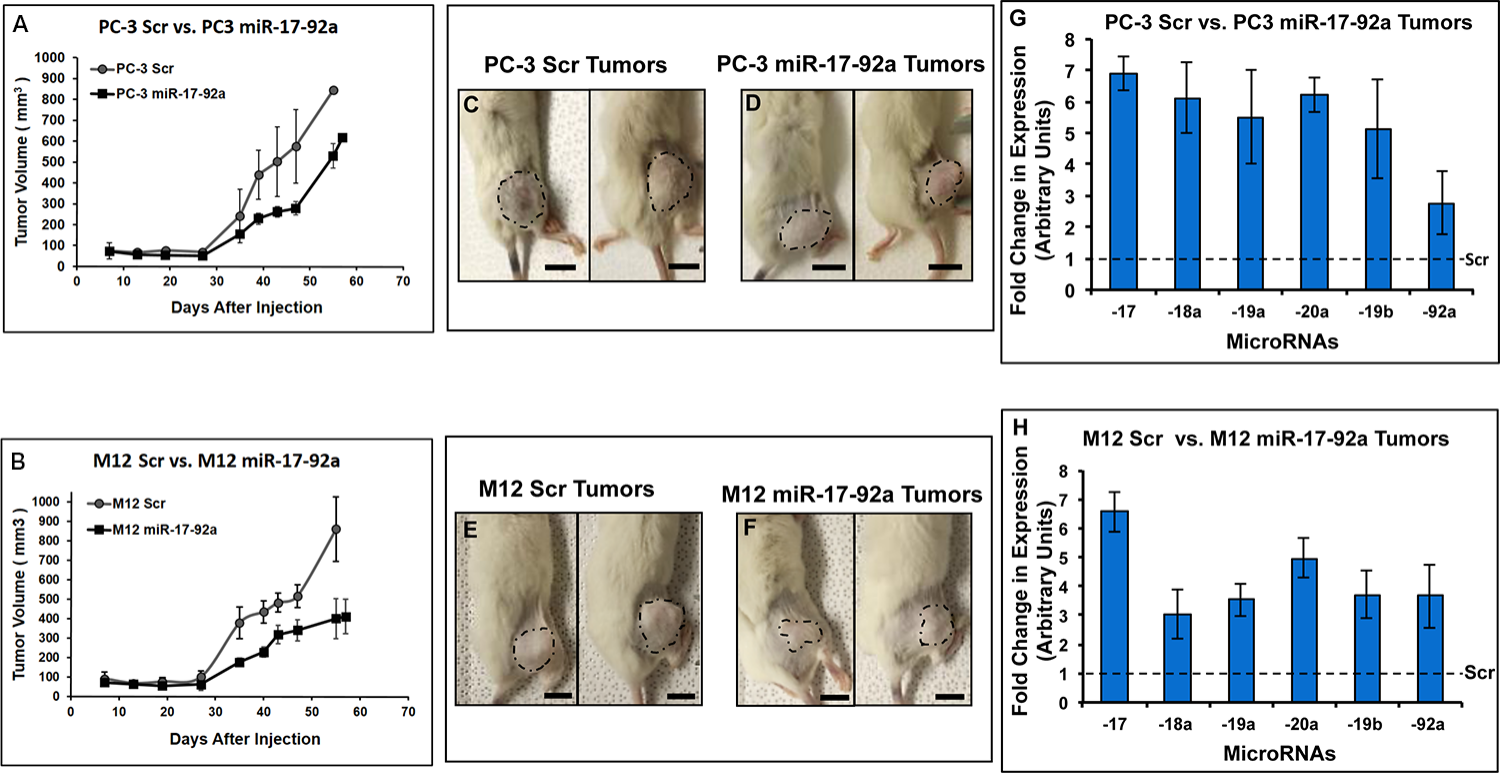
Expression of miR-17-92a cluster miRNAs reduced tumor growth in xenograft models Transfected sublines of PC-3 and M12 cells stably expressing miR-17-92a miRNAs or Scr RNAs were used to establish tumors in the flank of NSG mice. **A.** and **B.** Progression of tumor growth in mice. Mice were injected with PC-3 or M12 cells stably expressing miR-17-92a cluster miRNAs or Scr RNA and tumor growth monitored for 55-57 days by tumor volume measurement. **C-F.** Representative images of tumors in situ developed from PC-3 cells (C and D) orM12 cells (E and F). Scale bar: 10mm. **G.** and **H.** Quantitative PCR showing sustained overexpression of the miR-17-92a cluster miRNAs in tumors developed from PC-3 and M12 cells expressing miR-17-92a cluster miRNAs compared to the tumors developed from control cells expressing Scr RNAs (broken line across the X axis at the 1.0 value at the Y-axis). Data show mean expression ±SD of 4 animals/group.

We generated stable sublines expressing miR-17-92a miRNAs or Scr RNA of another metastatic prostate cancer cell line, M12 (Supplementary Figure S1B). Both PC-3 and M12 transfected sublines expressing miR-17-92a miRNAs or Scr RNAs were used for experiments on tumor growth in xenograft models. Cells were injected into the flanks of humanized NSG mice and tumor growth monitored for 55 days. The results showed a significant reduction in tumor volume (35%-53%) in mice injected with PC-3 and M12 cells expressing miR-17-92a cluster compared to the tumors in mice injected with cells expressing Scr RNAs (Figure 4A, 4B, 4C, 4D, 4E and 4F and Supplementary Figure S4). A delayed tumorigenesis was also noted in mice injected with prostate cancer cells expressing miR-17-92a cluster (Figure 4A). Quantitative RT-PCR Analysis of expression of the mature miRNAs in tumor tissues showed a sustained maintenance of the increased (>2.0 to >7.0 fold) expression of all cluster miRNAs (Figure 4G and 4H). In general, animals with both PC-3 and M12 miR-17-92 tumors survived longer than animals with tumors expressing Scr RNAs. Some of the M12 miR-17-92a tumor bearing animals were alive till 80 days post injection and showed sluggish or stunted tumor growth (data not shown).

### Expression of miR-17-92a inhibited cell migration and EMT

**Figure 5:**
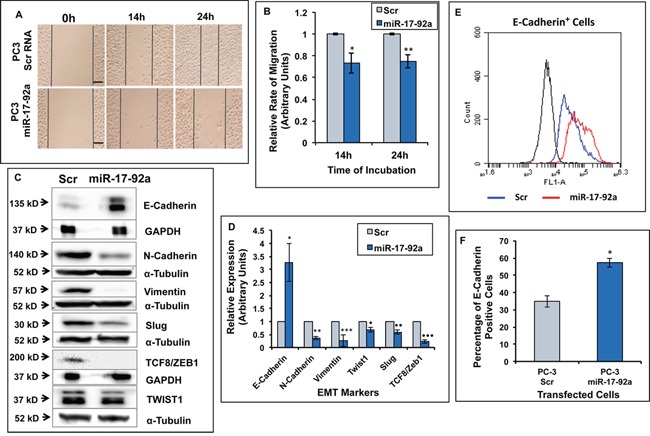
Expression of miR-17-92a cluster miRNAs inhibited migration and promoted an epithelial phenotype in prostate cancer cells Migration and expression of EMT markers were compared between PC-3 cells stably expressing miR-17-92a or scrambled RNA. **A.** and **B.** Migratory properties of cells were tested through wound healing assays at 14 and 24 hours after wound formation. The width of each wound was measured at three areas using light microscopy. Three wounds were made in each dish and the experiment was conducted in triplicate (A). Representative images of comparative migratory rates. Scale bar: 200μm. (B). Relative rates of migration at 14 and 24 hours. Data presented as the ratio of the distance traversed by the PC-3 cells expressing Scr RNA and miR-17-92a. Data represent mean±SD of three different experiments in triplicate wounds. *p=0.002; **p= 0.0004. **C-F.** Expression of miR-17-92a cluster miRNAs in PC-3 cells promoted a switch from EMT to MET phenotype. (C). Representative images of the immunoblots of EMT markers. GAPDH or α-Tubulin was used as internal controls. (D). Comparative analysis of EMT marker expression showing decreased expression of EMT markers but increased expression of the epithelial marker E-Cadherin. Values were normalized to internal controls. Data represent mean±SD of three separate experiments. *p=0.005; **p=0.00006; ***p=0.004; ·*p*=0.00002; ··*p*=0.001; ···*p=*0.00002. (E). Overlay of a two-parameter histogram of the cell population exhibiting surface expression of E-Cadherin. Result shows increased surface expression of E-Cadherin in PC-3 cells expressing miR-17-92a cluster miRNAs. (F). Quantification of the transfected PC-3 cells expressing E-Cadherin on the cell surface as determined by flow cytometry showing a 22% increase in the cells expressing E-Cadherin upon expression of miR-17-92a cluster miRNAs.

Previously, we noted a change in cell morphology upon restored expression of miR-17-92a miRNAs. To understand the contribution of miR-17-92a cluster expression to changes in cell behavior and phenotypes, we tested cell migration and expression of EMT markers such as, vimentin [51], Twist1 [52], Slug [53], TCF8/ZEB1 [54] and N-Cadherin [55] in PC-3 stable sublines expressing miR-17-92a miRNAs. The bright field images and quantification of the distance traversed by the cells in scratch assays after 14 and 24h of incubation showed a significantly slower rate (25%-27%) of migration upon expression of miR-17-92a compared to the control cells expressing Scr RNAs (Figure 5A and 5B). This observation was supported by the immunoblot assay results showing significantly reduced expressions of N-Cadherin, vimentin, Twist1, Slug, and TCF8/ZEB1 in these cells compared to the control cells (Figure 5C and 5D). Conversely, increased expression (3.2-fold) and surface localization (22%) of the epithelial maker E-Cadherin [56] was noted in PC-3 cells upon expression of miR-17-92a miRNAs as determined by western blots (Figure 5C and 5D) and flow cytometric analysis (Figure 5E and 5F). Our results demonstrate a phenotypic conversion of the highly aggressive PC-3 cells to a more adherent type of cells with a reduced migration rate.

### Expression of miR-17-92a cluster improved drug sensitivity of androgen-dependent and castration resistant prostate cancer cells

**Figure 6:**
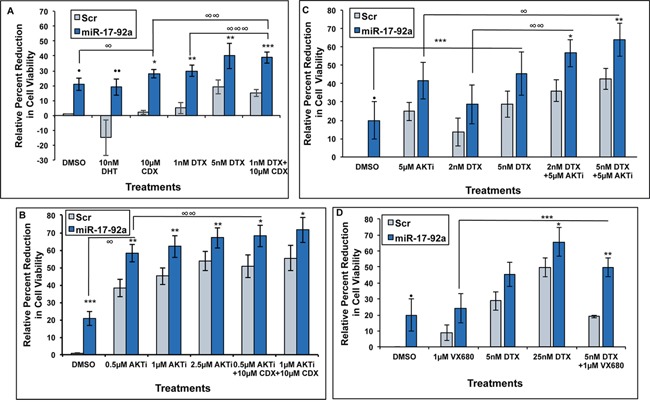
Expression of miR-17-92a cluster miRNAs increased drug sensitivity of prostate cancer cells Prostate cancer cells were transfected and treated with Casodex (CDX), docetaxel (DTX), pan-AKT inhibitor MK-2206 2HCl (AKTi), or Aurora kinase inhibitor VX-680. Sensitivity to individual or combinational treatments was measured through MTS assays. DMSO was used as the vehicle control. **A.** and **B.** Androgen sensitive LNCaP 104-S cells were transiently transfected and treated for 48 hours. (A). Percent reduction in viability of LNCaP cells expressing miR-17-92a miRNAs following treatment with 10μM DHT, 10μM CDX, 1nM DTX, 5 nM DTX, and combination of 1 nM DTX + 10μM CDX compared to cells expressing Scr RNAs. Data represent mean±SD of 4 independent experiments. ·*p*=0.0008; ··*p*=0.00003; *p=0.00001; **p=0.002; ***p=0.00003; and ****p=0.00001 (Scr vs. miR-17-92a transfected cells); ∞p=0.02 (DMSO vs. 10μM CDX; ∞∞*p*=0.004 *and ∞∞∞p*=0.016 (1 nM DTX+10μM CDX vs. 10μM CDX; and 1nM DTX+10μM CDX vs. 1 nM DTX, respectively) (B). Percent reduction in viability of LNCaP 104-S cells expressing miR-17-92a miRNAs following treatment with 0.5μM, 1μM, and 2.5μM AKTi alone or in combination with 10μM CDX compared to cells expressing Scr RNAs. Data represent mean±SD of 4 independent experiments. *p=0.01; **p=0.004; ***p=0.008; ·*p*=0.002; ··*p*=0.0006 and ···*p*=0.0003 (Scr vs. miR-17-92a transfected cells); ∞*p*=0.00002 and *∞∞p*=0.048 (DMSO vs. 0.5μM AKTi; and 0.5μM AKTi vs. 0.5μM AKTi+10μM CDX, respectively). **C.** and **D.** Androgen independent PC-3 cells were transiently transfected and treated for 72 hours. (C). Percent reduction in viability of PC-3 cells expressing miR-17-92a miRNAs following treatment with 5μM AKTi and 2nM DTX or 5nM DTX alone and in combination. Results show a significant reduction in viability of PC-3 cells only during combination treatments. Data show mean±SD of 3 independent experiments. *p=0.04 and **p=0.03 (Scr vs. miR-17-92a transfected cells); ***p=0.03 (DMSO vs. 5nM DTX); ∞*p*=0.04 (5μM AKTi vs. 5μM AKTi+5nM DTX); ∞∞*p*=0.018 (2nM DTX vs. 2nM DTX+5μM AKTi). (D). Percent reduction in viability of PC-3 cells expressing miR-17-92a miRNAs following treatment with 1μM VX680, 5nM DTX and 25nM DTX alone or in combination. Data show a significant reduction in viability of transfected PC-3 cells upon 25nM DTX alone and during combination treatment. Data represent mean±SD of 3 separate experiments. *p=0.01 and **p=0.02 (Scr vs. miR-17-92a transfected cells); ***p=0.03 (1μM VX680 vs. 5nM DTX).

Our previous studies showed down-regulation of miR-17-92a cluster miRNAs in LNCaP 104-S cells as they developed resistance to Casodex (CDX) [41]. Here we examined the involvement of miR-17-92a cluster in acquisition of CDX resistance of LNCaP 104-S cells. We used androgen-dependent LNCaP 104-S cells transiently transfected with pre miR-17-92a cluster DNA, and treated with CDX singly and in combination with the AKT inhibitor (AKTi) (MK-2206 2HCl), or docetaxel (DTX). As noted earlier, expression of miR-17-92a cluster miRNAs reduced the number of viable cells upon vehicle treatment compared to the Scr RNA expressing cells (Figure 6A). Viability assays after single and combination treatments showed a synergistic effect of the expression of miR-17-92a cluster and CDX treatment on the overall reduction of the number of viable cells when compared to Scr RNA expression (Figure 6A). Treatment with DHT (10nM) showed an increased number of metabolically active cells expressing Scr RNAs whereas expression of miR-17-92a miRNAs made these cells less responsive to DHT (Figure 6A). LNCaP 104-S cells expressing miR-17-92a miRNAs also showed an increased sensitivity to DTX at 1 nM and 5 nM concentrations compared to Scr control cells. Combination treatments with CDX and DTX at a lower (1 nM) concentration showed a significantly higher sensitivity than monotherapy (Figure 6A). Treatments with AKTi at 0.5, 1.0 and 2.5μM concentrations reduced cell viability significantly for both cell lines but to a much greater extent for cells expressing miR-17-92a (Figure 6B) compared to control cells expressing Scr RNAs. Combination treatments with CDX (10μM) showed a slightly higher reduction in mean percentage of cell viability. These results suggest a synergistic effect of both drugs (Figure 6B).

Next we examined the effect of miR-17-92a cluster on drug sensitivity of androgen-independent prostate cancer cells. Treatment of transiently transfected PC-3 cells expressing miR-17-92a cluster miRNAs or Scr RNAs with DTX and VX680 (Aurora kinase inhibitor) in combination showed a significant reduction in cell viability compared to control cells expressing Scr RNAs, but not when treated singly, except at a higher concentration (25nM) of DTX (Figure 6D). We tested a pan inhibitor of Aurora kinases as Aurora kinase inhibition, which causes mitotic arrest and apoptosis, has been recognized as an effective anticancer therapeutic strategy [57, 58]. Treatments with AKTi and DTX also showed an increased mean reduction in viability of PC-3 cells upon expression of miR-17-92a cluster miRNAs compared to control cells (Figure 6C) but not when treated singly suggesting a possible potentiating effect of miR-17-92a on the effectiveness of AKTi and CDX combination treatment. These observations indicate that restored expression of miR-17-92a cluster has a potential therapeutic benefit for treatment of both androgen-dependent and CRPC cells.

## DISCUSSION

In this study, we evaluated the functional significance of miR-17-92a cluster miRNAs in prostate cancer progression and resistance to chemotherapeutic agents. Because miRNAs are integrated into positive and negative regulatory loops of gene expression, it is important to study the expression and functions of all members of miR −17-92a cluster miRNAs concurrently to better mimic the *in vivo* environmental milieu. Our expression analyses provide evidence of an association of reduced expression of miR-17-92a cluster miRNAs with higher potential of biochemical failure, as indicated by higher CAPRA-S scores, of the majority of prostate cancer patients tested. To our knowledge, this is the first report on the expression of the miR-17-92a cluster in annotated prostate cancer clinical specimens.

Previously we showed loss of expression of all members of miR-17-92a cluster as prostate cancer cells developed resistance to ADT and antiandrogen CDX [41]. This observation opposes published studies showing increased expression of miR-17-92a cluster miRNAs in lung cancers and in B-cell lymphomas [13, 59]. The change in expression may either be driven by occasional amplification of the 13q31.3 locus and/or hyperactivation of *Myc* oncogene in these tumors, which is a potent transcriptional activator of *MIR17HG* gene [60]; alternatively, it may be due to increased stabilization of the mature miRNAs by endogenous regulatory RNAs or proteins. Here we noted a similar loss of expression of miR-17-92a in prostate tumors and in prostate cancer cells, which further confirms down-regulation of miR-17-92a cluster in prostate cancer. In support of our finding, reduced expressions of miR-17-5p and miR-17-3p in prostate cancer cell lines and tissues have been documented earlier [22, 23].

Our results revealed down-regulation of FGD4 and LIMK1 of Rho-GTPase signaling pathway; and cyclin D1 and SSH1 cell cycle regulatory proteins upon restored expression of miR-17-92a cluster miRNAs. A role of FGD4 in promotion of cancer cell migration has been reported earlier [44]. We also noted FGD4 overexpression in prostate tumors with higher Gleason scores and in androgen-independent tumors (unpublished observation). This observation suggests that expression miR-17-92a cluster facilitates establishing an anti-oncogenic and anti-migratory environment in prostate cancer cells by down-regulating FGD4, LIMK1, cyclin D1 and SSH1, as overexpression and oncogenic/metastatic functions of these proteins are documented in prostate and other cancers [44, 61–63]. The tumor suppressor function of miR-17-92a cluster in prostate cancer cells is supported by our results showing reduced cell proliferation and reduced phosphorylation/activation of ERK1/2 kinases and AKT in these cells. *In vivo* experiments also showed anti-tumorigenic and tumor growth inhibitory effects of miR-17-92a cluster, when expressed in highly aggressive PC-3 and M12 prostate cancer cells, which strengthen the inference that the miR-17-92a cluster exhibits tumor suppressor effects. Our study also demonstrates an anti-migratory function of miR-17-92a cluster that is substantiated by an increased expression of E-Cadherin and reduced expression of other mesenchymal markers. Migration inhibitory effects of miR-17-3p expression have been reported earlier in prostate cancer cells, which showed reduced vimentin expression upon of miR-17-3p overexpression [22].

Our study further establish miR-17-92a cluster as a potential therapeutic target, showing improved sensitivity of LNCaP 104-S and PC-3 cells to some of the drugs that are currently being used for the treatment of CRPC such as anti-microtubule (DTX), AKTi, anti-androgen (CDX) (androgen-dependent cells) and anti-Aurora kinases (androgen-independent cells). It can be speculated that the enhanced sensitivity of the miR-17-92a cluster expressing LNCaP 104-S cells to CDX is mediated through interfering androgen receptor function, as miR-17-5p has been shown to inhibit androgen receptor transcriptional activity through targeting p300/CBP [23]. The improved sensitivity of PC-3 cells expressing miR-17-92a cluster to DTX and VX680 in combination is presumably mediated through the inhibition of LIMK1 expression and activity (Figure 2 and Figure 3). Published studies showed that LIMK1 destabilizes microtubules [64] and interferes with DTX induced microtubule stability and active LIMK1 is needed for Aurora kinase A functional activation [65]. The enhanced sensitivity of both miRNA expressing cell types to AKTi singly and in combination with CDX (LNCaP 104-S) or DTX (PC-3) is possibly through miR-17-92a expression associated inhibition of AKT activation (Figure 3). However, this assumption does not rule out the possibility of alteration of other key signaling pathways and in-depth studies are required to understand the mechanistic aspects of miR-17-92a induced increased drug sensitivity.

Anti-proliferative, adhesive and anti-migratory properties of miR-17 upon overexpression in non-tumor cells and overall growth retardation and smaller organs in transgenic mice has been reported earlier [66] but our results show the maintenance of similar distinctive functions of the expressed miR-17 in the presence of expression of the other miRNAs of the cluster in prostate cancer cells. It is possible that because both miR-17 and −20a, which share the exact seed sequence but slightly different central region, are maximally expressed in the transfected cells and in tumors these two miRNAs may functionally synergize. It is clear that altered expression of specific targets decides the fate of a cell and the functional designation of a miRNA. Because of the intricacies of the complex regulatory network, and fine-tuning of gene expression by miRNAs and competing endogenous regulatory RNAs (ceRNAs), caution should be exercised to understand the function of miRNAs within the cellular context.

Taken together, our results strongly support the designation of miR-17-92a cluster miRNAs as tumor suppressors for prostate cancer and that replenishment of expression of miR-17-92a miRNAs as a cluster could be used for combination therapy with other frontline chemotherapeutic agents for treating advanced prostate cancers. Nonetheless, the reason for the loss of expression of miR-17-92a cluster miRNAs in prostate tumor is not clear, which could be a result of transcription silencing through promoter hypermethylation or enhanced destabilization of mature miRNAs. This assumption is further attributable to the fact that c-MYC expression does not corroborate with the variable expression of miR-17-92a in BPH-1, LNCaP 104-S and PC-3 cells. c-MYC expression was noted to be similar in BPH-1 and LNCaP 104-S cells but higher in PC-3 cells (Supplementary Figure S5). This discrepancy indicates a possible uncoupling of the mechanisms of down regulation of miR-17-92a miRNA cluster and expression of c-MYC in prostate cancer cells. In depth studies are required to understand the mechanistic roles of miR-17-92a cluster in modulation of particular pathways that lead to inhibition of cell proliferation and increased drug sensitivity.

## MATERIALS AND METHODS

### Acquisition of patient tissues

Prostate tissues obtained by radical prostatectomies were procured in the Cooperative Human Tissue Network (Southern division) at the University of Alabama at Birmingham (UAB) in accordance with an approved IRB protocol. Patients were selected based on the low, medium and high risk of biochemical failure as predicted from the pathological report and the medical record to determine the risk factor based on the CAPRA-S score [42]. Formalin-fixed paraffin embedded prostate tissues were macrodissected separately for tumor areas and for matching uninvolved areas and used for RNA extraction followed by qRT-PCR of the miR-17-92 miRNAs.

### RNA extraction and quantitative real-time PCR

Total RNA from prostate tissues or cell lines was extracted using RecoverAll (ThermoFisher) or Cells-to-Cts kit (System Biosciences). Total RNA was converted to cDNA utilizing the QuantiMir RT Kit (System Biosciences) in which small RNAs were tagged with poly-A tail first followed by conversion to cDNAs using oligo-dT primers. Expression of matured miRNAs was determined by quantitative real-time PCR using specific primers (System Biosciences) (Supplementary Figure S6) for all members of the miR-17-92a cluster and 3 internal control snRNAs, and SYBR green PCR reagents (ThermoFisher). MicroRNA IDs listed in the text are based on Sanger miRBase identifiers. Primers were designed to maintain uniform amplification efficiencies. Quantitative RT-PCR was conducted using the Applied Biosystems 7900HT thermal cycler and data analyzed using SDS2.3 software. DNA concentrations were assessed through SYBR Green fluorescence and normalized to that of the passive reference dye, ROX [41]. Ct values generated by the SDS2.3 software were normalized according to the average Ct values of the three internal controls (System Biosciences) using qbasePLUS software (Biogazelle). The Ct values of all miRNAs were normalized to these controls and the relative expression values were then generated using qbasePLUS software. The Ct values were used to derive ^ΔΔ^Ct values using the miRNome analysis software (System Biosciences).

### Cell lines and transfection

PC-3 prostate cancer cells obtained from ATCC were cultured in F12 HAM containing 10% fetal bovine serum (FBS) and 1% antibiotic/antimycotic (Invitrogen). M12 prostate cancer cells (a gift from Jay Ware) [67] were cultured in RPMI containing 5% fetal bovine serum, 10ng/mL EGF, ITS mix (5μg/mL insulin, 5μg/mL transferrin, 5ng/ml selenium), 50μg/mL gentamycin, and 0.1μM dexamethasone. The androgen responsive LNCaP 104-S cells were a generous gift from Dr. Shutsung Liao (University of Chicago). This androgen dependent LNCaP subline was isolated from the parental LNCaP cells and characterized as previously described [68]. LNCaP 104-S cells were maintained in DMEM containing 10% fetal bovine serum, 1nM DHT (Sigma-Aldrich) and 1% antibiotic/antimycotic. For ectopic expression of pre-miRNAs of miR-17-92 cluster, PC-3, M12 and LNCaP 104-S cells were transfected with a modified expression vector containing a precursor clone of miR-17-92 cluster PMIRH 17-92PA-1 (System Biosciences) using Lipofectamine 3000 (Life Technologies, ThermoFisher). The vector was engineered for replacing the DNA for GFP between Not1 and Sal1 sites with a cassette containing Neomycin^r^ gene driven by SV40 promoter (pCMV6-AC-GFP, Origene,) Stable clones of PC-3 and M12 cells expressing miR-17-92 cluster pre-miRNAs were selected using G418 and used for subsequent experiments. PC-3 and M12 cells expressing scrambled RNA were also generated as the control cell lines. Stable sublines were used for xenograft model experiments and other specific experiments. LNCaP 104-S and PC-3 cells were transiently transfected with the vector containing DNAs for miR-17-92a precursor RNAs or the scrambled RNA and used for specific experiments for drug sensitivity assays using MTS based CellTiter Aqueous One Solution cell proliferation assay kit (Promega). Transfected cells were treated with various dilutions of inhibitors singly or in combination and incubated for 48-72 hrs before monitoring cell proliferation.

### Western blotting

Lysates of PC-3 cells expressing either miR-17-92 miRNAs or scrambled RNA were prepared and used for immunoblotting with antibodies specific for FGD4 (N1N3) (Genetex), LIMK1 (BD Biosciences), cyclin D1 (ThermoFisher), GAPDH (Sigma Aldrich) and alpha-tubulin (Sigma-Aldrich). Antibodies specific for SSH1, phospho-Cofilin1, phospho-AKT, phospho-p44/42 MAPK, phospho-PRAS40, E-Cadherin, N-Cadherin, vimentin, Slug, TCF8/ZEB1 and TWIST1 were obtained from cell signaling.

### Dual label immunofluorescence

PC-3 cells stably expressing miR-17-92a miRNAs or scrambled RNA were seeded on poly-l-lysine coated coverslips. The next day, cells were fixed with 4% paraformaldehyde, probed with antibodies specific for pERK1/2 or Ki-67 (cell signaling). Cells were then stained with goat anti-mouse Alexa-Fluor 488 and goat anti-rabbit Alexa-Fluor 561 secondary antibodies. Cells were visualized with a Leica SP5 confocal microscope and images were analyzed using Leica LAS AF software suite.

### Luciferase reporter assays

A 3′UTR-luciferase reporter vector containing the first 206 bases of the FGD4 3′UTR (HmiT002135-MT01) along with the control vector (CmiT000001-MT06) were obtained from GeneCopoeia. The mutant FGD4 3′UTR was generated by amplifying the HmiT002135-MT01 vector with the following primer sequences: Forward Primer 5′-GCTCTTCTTACA CATCTGCTAGCCAGTTATGTTGAAAAATATAGG-3′, Reverse Primer 5′-CCTATATTTTTCAACATAACTGGCTAGCA GATGTGTAAGAAGAGC-3′. The luciferase reporter constructs containing the WT-FGD4 3′UTR, Mut-FGD4 3′UTR, or a control 3′UTR were transiently transfected into PC-3 cells using X-tremeGENE HP (Roche). Luciferase activity was monitored 48hrs post transfection using the Luc-Pair Duo luciferase assay kit from GeneCopoeia. The firefly luciferase activity was normalized to the Renilla luciferase activity for each well.

### Experiments using xenograft model

Xenograft experiments were conducted in 6-8 weeks old male NSG (NOD.Cg-PrkdcscidIl2rgtm1Wjl/SzJ (005557)) mice obtained from Jackson Laboratory, and maintained and treated under specific pathogen-free conditions. Stable pool of PC-3 and M12 Cells (2 × 10^6^/mouse) suspended in 0.1% matrigel in 100μl volume were injected subcutaneously into the flank and tumor growth monitored for 55 days. Tumor growth was assessed by measurements with a caliper and volume was calculated as 0.52 × length × width × 2 as tumor grew. Tumors were harvested after the specified time, tissue architecture examined by H&E staining of formalin fixed paraffin embedded sections and used for RNA extraction for qRTPCR of the matured miR-17-92a miRNAs expressed in the tumor. Xenograft experiments were performed according to an animal protocol approved by the Animal Care and Use Committee of the University of Central Florida.

### Wound healing assay

PC-3 cells grown in HAM-F12K containing 5% FBS, were grown to confluency (24-36hrs after seeding) and wounds were created using a micro pipet tip. The cells were rinsed twice with media and incubated in media containing 5% FBS at 37°C. Wound healing within the scratch was observed at 0, 14 and 24 hrs. Images of the scratch were taken under 10x magnification using a Nikon eclipse TE200 inverted microscope paired with Nikon elements F 2.20 software. The average width of each scratch was determined using ImageJ software. The relative rate of migration was determined through the initial analysis of the remaining width of the scratch at different times and subtracting that from the scratch width at 0hr. Next, the ratio of the distance traversed by the individual cell lines was determined.

### Flow cytometry

PC-3 cells expressing miR-17-92a cluster miRNAs or scrambled RNA were seeded and allowed to grow to 80% confluency in complete growth medium. Cells were dissociated using 10mM EDTA in PBS, washed with PBS and fixed with 4% paraformaldehyde. The fixed cells were blocked with PBS containing 10% goat serum, followed by incubation with E-Cadherin antibodies on ice for 1 hour. The cells were then washed in PBS, followed by incubation with AlexaFluor488 goat anti-rabbit antibodies (Life Technologies). Labeled cells were washed again in PBS containing 0.05% Tween-20. The labeled cells were detected using the BD Accuri flow cytometer and analyzed with the BD CSampler software. Threshold of positive staining was set above the intensity of cells incubated with control antibodies.

### Statistical analysis

All statistical analyses were performed using Student t-test or one-way ANOVA for independent measures using GraphPad Prizm.

## SUPPLEMENTARY MATERIALS TABLE AND FIGURES


