# Supplementary material for: The other face of miR-17-92a cluster, exhibiting tumor suppressor effects in prostate cancer
Source: Oncotarget. 2016 Sep 16;7(45):73739–53. doi: 10.18632/oncotarget.12061 (PMC5340125; doi:10.18632/oncotarget.12061)
Supplement: Supplementary file 1 [file oncotarget-07-73739-s001.pdf]

The other face of miR-17-92a cluster, exhibiting tumor suppressor effects in prostate cancer

SUPPLEMENTARY FIGURES AND TABLE

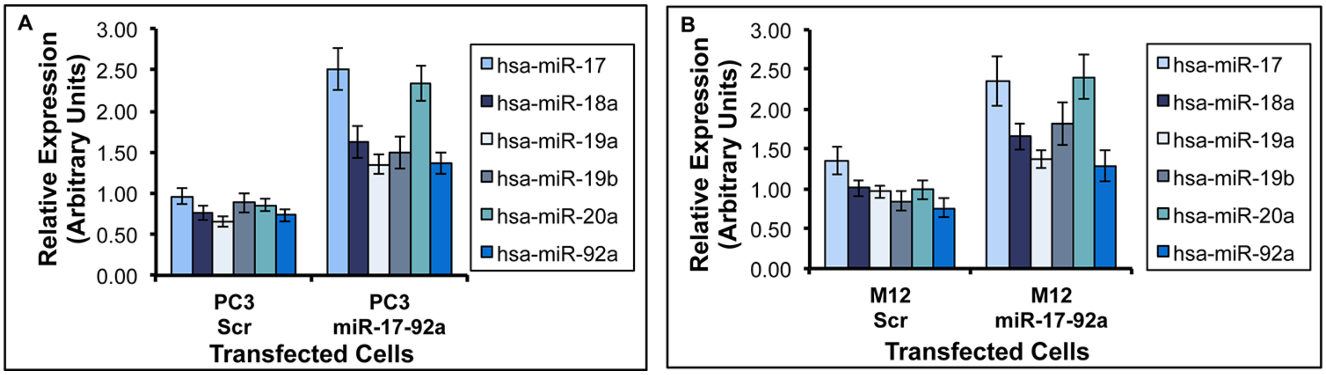

Supplementary Figure S1: Quantitative PCR analysis of the expression of the members of the miR-17-92a cluster in stably transfected PC-3 and M12 cells expressing miR-17-92a miRNAs of Scr RNA. Data represent mean±SD of 3 or more independent analyses.

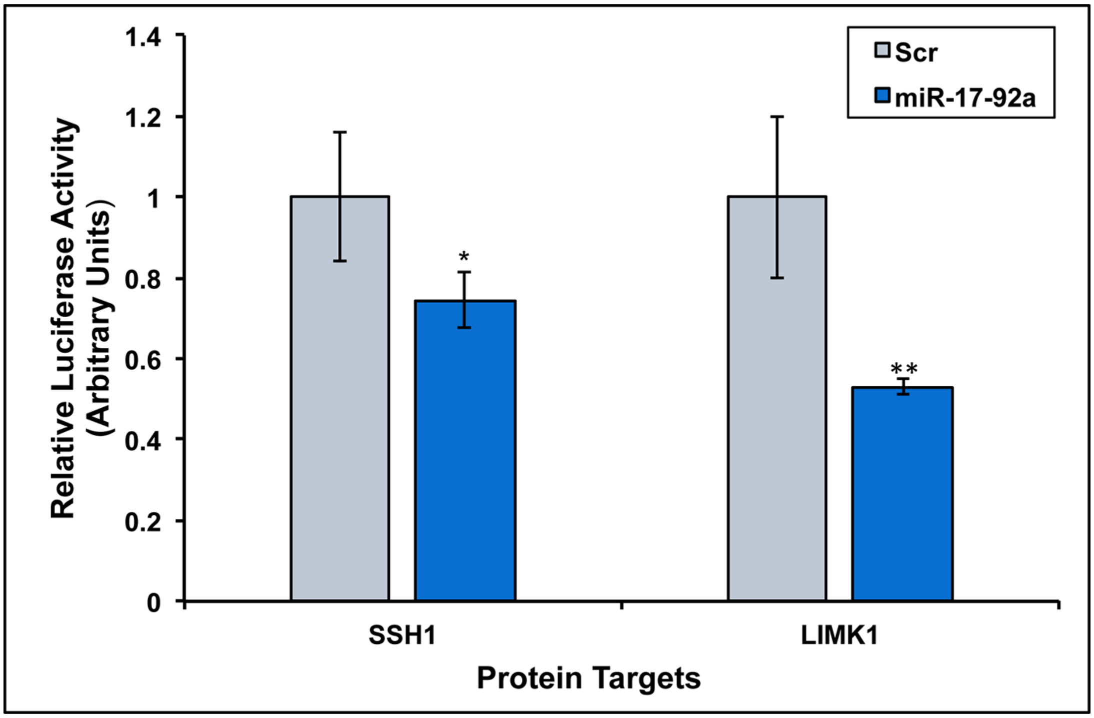

Supplementary Figure S2: Luciferase reporter assays confirming that miR-17a and -20a directly target SSH1 and LIMK1 through binding to the specified seed sequence at the 3'UTR. PC-3 cells were co-transfected with the luciferase constructs containing a nonspecific DNA sequence, a fragment of wild type SSH1 or LIMK1 3' UTRs along with a plasmid expressing either miR-17-92a miRNAs or scrambled RNA. Data represent the mean±SD of three independent experiments. *p=0.003 (Scr vs. SSH1); **p=0.00001 (Scr vs. LIMK1). Methods: The luciferase reporter constructs containing the WT-SSH1 and -LIMK1 3'UTR were generated by cloning the 3'UTR regions of these genes. The gene fragments of SSH1 and LIMK1, 836 bp and 984 bp respectively, were PCR amplified using the primers (*Ssh1:* Forward 5'-GTAGACAGGAGTCCCGATAAG-3', Reverse 5'-TCACAAGAAGCACACACAC3'; *Limk1:* Forward 5'-GTAGACCGCTTCCCCTGC-3', Reverse 5'-CCTCCCTAAGTCATGGTCCC-3') and genomic DNA from BPH-1 cells as the template using standard PCR amplification conditions. The amplicons were first ligated into the TA cloning vector pGEM-T Easy, before being excised through digestion with restriction enzymes AccI and NotI. The amplicons were cloned into the pMirGlo plasmid (Promega) at AccI (7339 bp) and NotI (93 bp) sites.

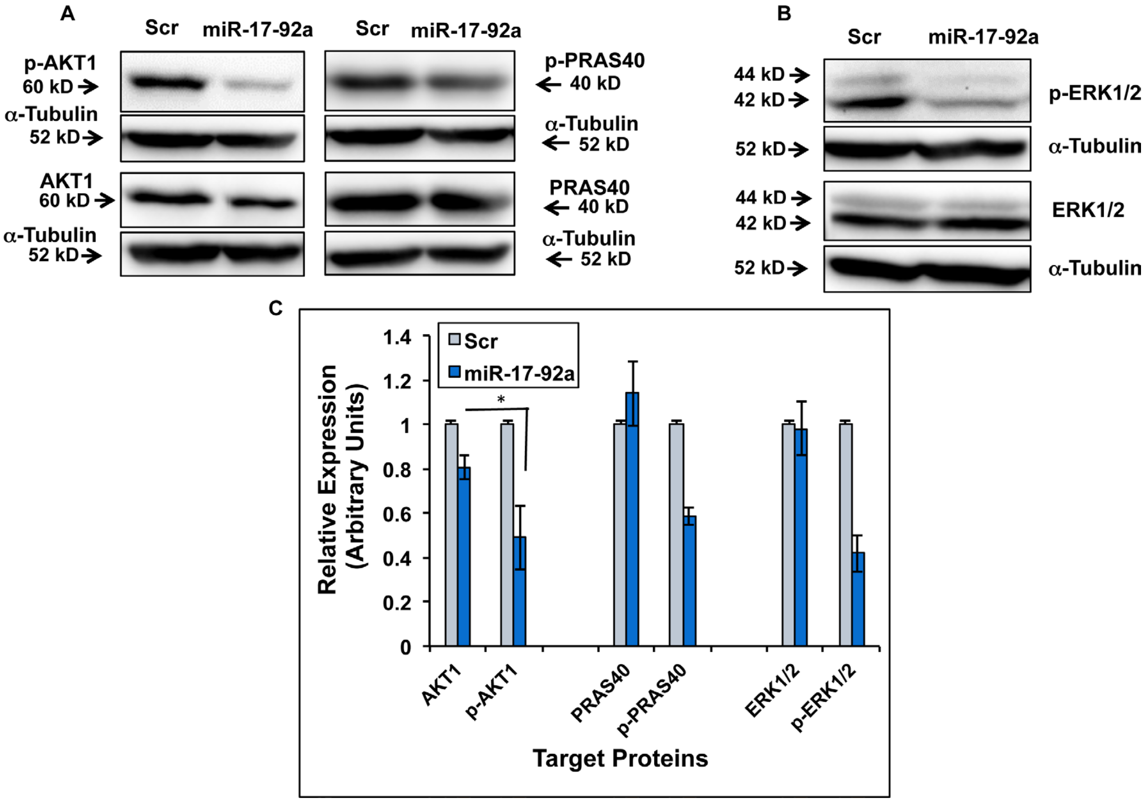

Supplementary Figure S3: Expression of miR-17-92a miRNAs reduced activation of MAPK and AKT pathways. A and **B.** Immunoblot analysis showing phosphorylated and total AKT, -PRAS40 (AKT substrate) and -ERK1/ERK2, in lysates from PC-3 cells stably expressing miR-17-92a cluster compared to cells expressing Scr RNAs. Antibodies α-tubulin was used as the loading controls. Data shows reduced phosphorylation of AKT, PRAS40 and ERK1/2 but no significant change in the total proteins. **C.** Densitometric analysis of thephosphoprotein and total protein concentrations normalized to internal controls. Data represent mean±SD of three separate experiments Methods: Total cell lysates were used for immunoblot analysis using primary antibodies for both phosphorylated and total proteins from Cell Signaling (total and phospho-p44/42 MAPK ERK1/2, total and phospho-PRAS40 and phospho-AKT). Antibodies for total AKT was obtained from Transduction Laboratories. Data represent mean ±SD of 3-4 separate experiments. **p*=0.006 (Total AKT and phospho-AKT).

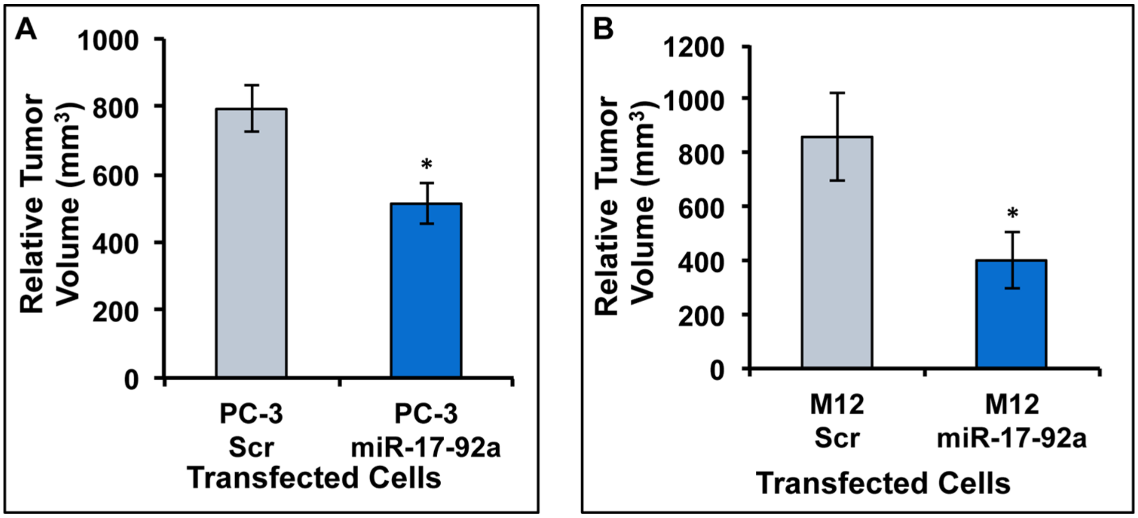

Supplementary Figure S4: Comparison of tumor volumes at 55 days post injection for PC-3 cells. A. or M12 cells **B.** Data represent mean tumor volume±SD of 4 animals/group. **p*=0.002 (A); ***p*=0.003 (B).

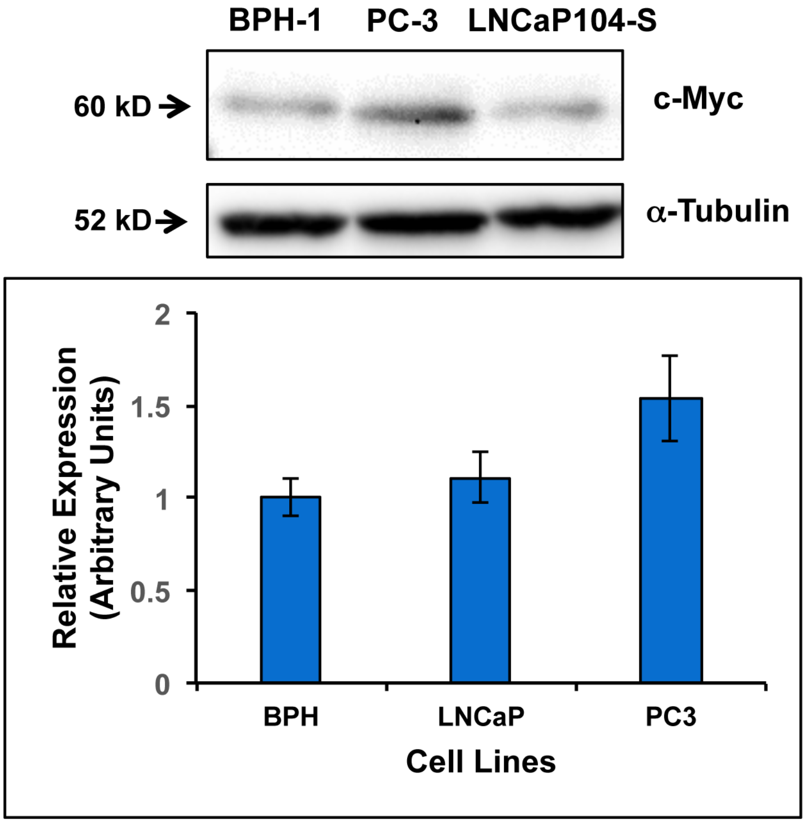

Supplementary Figure S5: Expression of c-MYC in prostate cell lines. A. Immunoblot analysis showing c-MYC expression in BPH-1, LNCaP 104-S and PC-3 cells. Expression of α-tubulin was used as the loading controls. **B.** Densitometric analysis of c-MYC expression in LNCaP 104-S and PC-3 cells compared to BPH-1. Data show a higher expression of c-Myc in PC-3 cells compared to BPH-1 and LNCaP 104-S cells. Expression of α-tubulin was used for normalization of the individual expression. Data represent mean±SD of three separate experiments. Methods: Total cell lysates were used for immunoblot analysis using primary antibodies for c-Myc (Cell Signaling).

Primer Sequence for qRT-PCR	
miR-17	CAAAGUGCUUACAGUGCAGGUAG
miR-18a	CAAAGUGCUUACAGUGCAGGUAG
miR-19a	UGUGCAAAUCUAUGCAAAACUGA
miR-19b	UGUGCAAAUCCAUGCAAAACUGA
miR-20a	UAAAGUGCUUAUAGUGCAGGUAG
miR-92a	UAUUGCACUUGUCCCGGCCUGU

Supplementary Figure S6: Primer sequences used for quantitative PCR amplification of miR-17, miR-18a, miR19a, miR-20a and miR-92a.

Supplementary Table S1: miRNA expression pattern in tumor tissues

miRNAs	Down-regulated/ undetectable	Up-regulated	Loss in percent of cases
miR-17	15	11	58
miR-18a	16	10	62
miR-19a	16	10	62
miR-19b	16	10	62
miR-20a	18	8	69
miR-92a	18	8	69
